# Is the standardized antibiotic administration ratio (SAAR) ready for prime time?

**DOI:** 10.1017/ash.2023.186

**Published:** 2023-07-17

**Authors:** Yi Guo, Elizabeth Dodds Ashley

**Affiliations:** 1 Department of Pharmacy, Montefiore Medical Center, Albert Einstein College of Medicine, Bronx, New York, USA; 2 Division of Infectious Diseases & International Health, Duke University School of Medicine, Durham, NC, USA

## Abstract

The standardized antibiotic administration ratio (SAAR) enables comparison of antibiotic use (AU) within and between hospitals and identifies target locations and antimicrobials for stewardship interventions. Thousands of institutions have already been submitting AU to the National Healthcare Safety Network. We highlight the benefits and meaningful utilization of SAAR in conjunction with antimicrobial stewardship interventions to improve antimicrobial prescribing in the clinical setting.

## What is the standardized antibiotic administration ratio (SAAR) and why is it necessary?

Unlike common infection prevention metrics, where the goal is no harm, it has been more challenging to set targets for antimicrobial use (AU) without access to external, risk-adjusted data on AU in like patient populations. In 2015, the Centers for Disease Control and Prevention (CDC) introduced the SAAR within the antimicrobial use and resistance (AUR) module, a domain of the patient safety component of the National Healthcare Safety Network (NHSN).^1^ The SAAR enables comparison of AU within and between hospitals, and it identifies target locations and antimicrobials for stewardship interventions to reduce excess use.^2^

The SAAR is a ratio of actual to expected AU.^2^ It is calculated based on the nationally aggregated adult and pediatric data with risk adjustment according to specific patient-care locations, facility type, hospital teaching status, hospital bed size, number of intensive care unit beds, average facility length of stay, etc.^3^ Although the SAAR does not indicate whether the AU is appropriate per indication, dosing schema, or duration, it is the most direct tool for tracking AU patterns and identifying areas of outlier antibiotic use for possible improvement. Tracking the SAAR over time can provide a measurement of the effectiveness of an antimicrobial stewardship program (ASP).^4^

## What is at stake?

The CDC prioritizes AU tracking and reporting to NHSN to achieve compliance with the core elements of hospital ASP.^5^ As of August 2022, > 2,400 acute care hospitals have voluntarily reported to NHSN AUR module.^6^ The CDC statistical models used to calculate the SAAR benchmark were updated using 2017 data and have become more robust as an increasing number of institutions submit AU data to NHSN since the inception of the AUR module.^2^ In September 2022, the AUR module was further refined by incorporating the Targeted Assessment for Stewardship (TAS) to provide the AU-cumulative attributable difference (AU-CAD) value which translates the SAAR target into a tangible number of antimicrobial days to reduce or add to achieve a desired SAAR.^7^ These new data can further fine-tune ASP-targeted interventions and can aid with goal setting, including determining required staffing levels for interventions based on the volume of work required.^7^

On August 10, 2022, the Centers of Medicare and Medicaid Services (CMS) announced the final ruling (section IX.H.5.b.) of the updated Public Health and Clinical Data Exchange Objective, which adds the AUR measure to the 4 required reporting domains starting with the CY 2024 reporting period.^8^ This requires the purchase of an electronic platform through a third-party vendor or the development of an AUR reporting solution within the electronic health records. As AUR reporting becomes a requirement, an estimated median cost of $187,400 is needed to build and maintain the AUR submission system per institution.^8^ However, these costs are far outweighed by the >$4.6 billion in healthcare costs spent annually treating antibiotic resistance threats as well as significant financial penalties faced by hospitals if they are noncompliant with AUR submission process by 2024.^8,9^ Currently, the AUR submission requirement is pay for reporting rather than performance, and public reporting of antibiotic use data at the facility level is not planned. The reporting is meant to provide all antimicrobial stewards with a common, actionable, risk-adjusted metric for improving antibiotic use.

Although the upfront costs can be significant for some institutions, the benefits of AU tracking and reporting coupled with ASP interventions can lead to cost savings by potentially reducing excess antimicrobial adverse consequences, like *C. difficile* infection. For example, in our own experience we realized a cost saving of $500,000 due to reduced antibiotic use with a series of stewardship interventions, including AU provider feedback reports, at the Montefiore Health System in 2022 (internal data).

## What can you do with AUR data?

**Figure 1.**
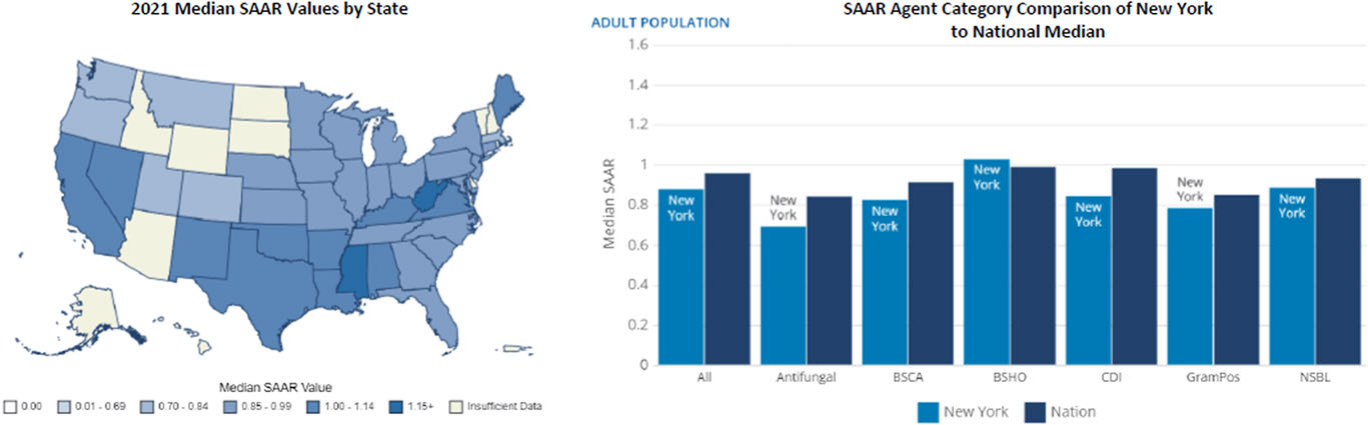
Inpatient antibiotic use by standardized antibiotic administration ratio (SAAR). Note. BSCA, broad-spectrum antibacterial agents for community-acquired infections; BSHO, broad-spectrum antibacterial agents for hospital-onset infections; CDI, antibacterial agents posing the highest risk for *Clostridioides difficile* infection; GramPos, antibacterial agents for resistant gram-positive infections; NSBL, narrow-spectrum beta-lactam agents. https://arpsp.cdc.gov/profile/inpatient-antibiotic-use/all; https://arpsp.cdc.gov/profile/geography/new-york?phenotype-select-ar-by-state=arPhenotype15

**Figure 2.**
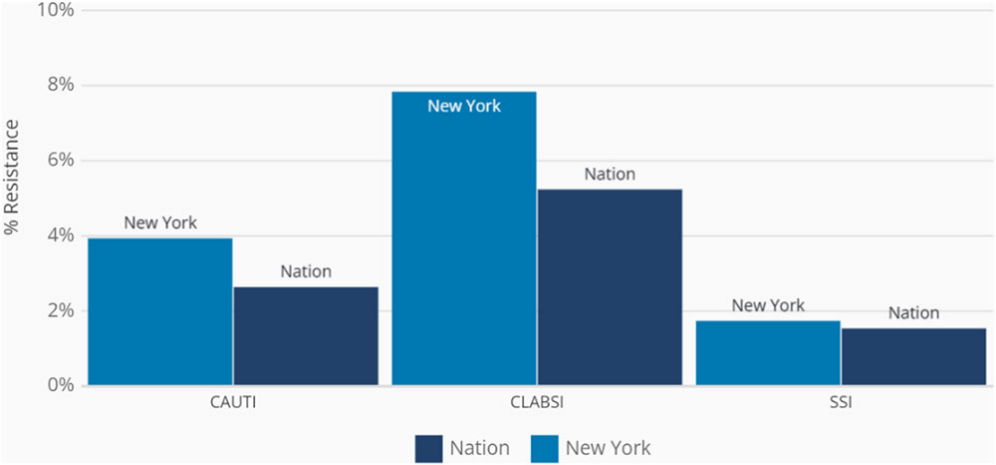
Percentage of carbapenem-resistant Enterobacterales as healthcare-associated infection type in New York compared to the nation in 2020. Note. CAUTI, catheter-associated urinary tract infection; CLABSI, central line-associated bloodstream infection; SSI, surgical site infection. https://arpsp.cdc.gov/profile/geography/new-york?phenotype-select-ar-by-state=arPhenotype15

The goal of AUR is to enable regional and national comparison of AU in context of NHSN antibiotic resistance and healthcare-associated infection data to provide a comprehensive risk assessment for the healthcare consumer.^10,11^ Importantly, this will provide more actionable stewardship data to frontline stewards. Having universal data submission from all US hospitals will facilitate better, more inclusive risk adjustment models going forward. Figure 1 displays 2021 median SAAR values by state for all SAAR agents hospital-wide. Several states from the West, Southwest, and Southeast regions have a SAAR >1. Although the overall SAAR is reported as < 1 for some states, individual SAAR categories may still be above the national median in these regions. For example, the overall reported median SAAR for New York State is 0.874. However, the SAAR for broad-spectrum hospital-onset agents is > 1, which is above the national median (Figure 1).^10,11^ Moreover, higher rates of antibiotic resistance are observed for hospital-acquired infections (HAIs), such as catheter-associated urinary tract infection, central line-associated bloodstream infection, and surgical site infection, for New York compared to the national data (Figure 2).^11^ AUR data may provide additional insights into both the cause and consequence of excess HAIs caused by drug-resistant pathogens. Therefore, the SAAR enables infection control and prevention (ICP) and ASP to comprehensively review their data using an integrated approach and “speak the same language” in terms of benchmarked, risk-adjusted NHSN data.

Decreasing HAIs is a mutual goal of ASP and ICP. Receiving antibiotics is one of the highest risk factors for developing hospital-onset *C. difficile* infection (HO-CDI).^12^ At Montefiore Medical Center (MMC) in the Bronx, New York, the SAARs for agents predominantly used for broad-spectrum hospital-onset infections and agents posing the highest risk of CDI are closely monitored. The ASP team reviews AU for the units where the SAARs indicate that AU is above expected rates on a monthly basis. Moreover, in collaboration with ICP, the ASP team also provides periodic feedback on each HO-CDI case, auditing antibiotic appropriateness in the 30 days prior to HO-CDI onset. These findings were shared with provider teams to improve local antibiotic use. Hence, the synergized efforts of ASP and ICP can optimize NHSN metrics leading to improved patient outcomes.

## How can the SAAR complement other stewardship data?

Locally, AU data allow stewards to develop interventions targeted at optimizing AU and to evaluate their effectiveness. Livorsi et al.^13^ showed that SAARs are useful in monitoring multifaceted ASP activities, and SAARs declined across multiple categories with efforts to encourage providers to prescribe more narrow-spectrum antimicrobials and to stop antimicrobials when they are not indicated. Numerous published data suggest that tracking SAAR categories allowed the ASP to reduce inappropriate quinolone, aztreonam, and carbapenem use.^14^ SAAR is a useful tool for identifying stewardship targets and monitoring facility-level changes in AU over time.

Provider performance reports on comparative antibiotic utilization are among the most powerful motivating tools in stewardship, a potential additional application of the SAAR.^2,4,15^ Shealy et al.^15^ conducted a prospective cohort study on the utility of SAAR reporting and inter-facility comparisons as a motivational tool. Routine sharing of these data resulted in significant reductions in overall and broad-spectrum AU across a 3-hospital system.

Similarly, at MMC, ASP encourages sharing SAAR reports and best practices by affiliates at the health-system antimicrobial council meeting. The ASP team also disseminates the monthly SAAR reports and audit results of AU and DOT with stakeholders across multiple disciplines (pediatrics, critical care units, etc.) to promote judicious use of antimicrobials. These 2-way dialogues not only empower stakeholders to be good stewards but also enable further opportunities for ASP collaboration in the future. In the Duke Antimicrobial Stewardship Outreach Network, health systems including hospitals of disparate sizes and patient populations have converted to the SAAR for internal tracking by senior leadership. Similarly, these data can be used for more widespread comparisons, as was described by the Antimicrobial Stewardship Collaborative of South Carolina, where statewide reports highlighting areas of use compared to peers were prepared and distributed.^16^

Lastly, observed-to-expected ratios are already in a language that health-system administrators use in multiple other domains. Thus, antibiotic use data using the SAAR ratio should be easy to convey to administrators, particularly when demonstrating program effect.

### Future state of the SAAR

Thanks to the national prioritization of antimicrobial stewardship and combatting drug resistance, hospital antimicrobial data tracking and reporting have made significant strides over the past decade. The development, uptake, and progress of NHSN AUR option continue to evolve. Moreover, millions of dollars are at stake for hospitals given the recent updates to the CMS promoting interoperability program.^8^ The introduction of TAS with AU-CAD values provides new tangible data to improve ASP interventions.^7^ Future enhancements to electronic data reporting capacity in NHSN are being planned that will eventually permit the flow of patient-level data for AU, further refining the data available to hospitals and its value and applicability.^17^ In summary, with national prioritization of antimicrobial stewardship, associated financial incentives to hospitals, and ongoing refinement of data available to stewardship programs and clinical stakeholders, this is the appropriate time for programs to commence their AUR journeys and adopt the SAAR if they have not already done so.
